# Editorial: Proceedings of the Inaugural ISESSAH Conference

**DOI:** 10.3389/fvets.2019.00366

**Published:** 2019-10-18

**Authors:** Bouda Vosough Ahmadi, Jonathan Rushton, Barbara Häsler, Henk Hogeveen, George John Gunn

**Affiliations:** ^1^The European Commission for the Control of Foot-and-Mouth Disease (EuFMD), Food and Agriculture Organization of the United Nations (FAO), Rome, Italy; ^2^Institute of Infection and Global Health, University of Liverpool, Liverpool, United Kingdom; ^3^Royal Veterinary College (RVC), University of London, London, United Kingdom; ^4^Business Economics Group, Wageningen University & Research, Wageningen, Netherlands; ^5^Scotland's Rural College (SRUC), Edinburgh, United Kingdom

**Keywords:** economics of animal health, social sciences of animal health, ISESSAH, animal diseases, animal health policies

Tackling the challenge of supplying sufficient food for the increasing human population in an environmentally and ecologically sustainable manner is an important focus of public and private-sector policy makers. Livestock production chains play crucial roles in fulfilling the global sustainable development goals defined by the United Nations (1). Animal diseases and animal welfare problems are considered as major barriers in achieving optimized production and profit levels where ecological and environmental damages are minimized. Emerging and reemerging transboundary diseases that are often highly contagious [such as foot-and-mouth disease (FMD), peste des petits ruminants (PPR), African swine fever (ASF), and highly-pathogenic avian influenza (HPAI)] continue to threaten livestock industries in both developed and developing counties. Similarly, other infectious and non-infectious diseases continue to impose a socioeconomic burden on food production chains and on the wider social economy in many countries.

To tackle animal diseases, resolve animal welfare problems, and mitigate their environmental and socioeconomic burden, continuously supplied quantitative and qualitative socioeconomic research is crucial to support policy-making process. To achieve this goal, the International Society for Economics and Social Sciences of Animal Health (ISESSAH) was established in 2017 and its inaugural conference was held in March 2017 in Avimore, Scotland. The current Research Topic is devoted to 11 papers from among those presented in the first ISESSAH conference. Papers presented in this Research Topic cover broad but relevant areas of focus that can be grouped into three main categories: (1) economic assessment and managerial decision analysis of production diseases; (2) economic and policy assessment of contagious transboundary animal diseases and zoonoses; and (3) human behavior in relation to animal health and market analysis.

## Economic Assessment and Managerial Decision Analysis of Production Diseases

Romero et al. assessed the financial impact of subclinical mastitis in dairy farms in Colombia. The authors showed that mastitis imposed a greater financial loss on small and medium-sized dairy farms than on larger farms, and they highlighted the gap in our understanding of the costs and effectiveness of on-farm intervention measures. Niemi et al. reported the costs of postpartum dysgalactia syndrome (PPDS) and locomotory disorders of sows due to their impacts on productivity and replacement rates in Finland. Using a stochastic dynamic programming model that maximized the return on sow space unit, they demonstrated that PPDS and locomotory disorders imposed financial losses of €29.1 and €11.5 per housed sow, respectively, during her lifetime.

By conducting a literature review, consulting experts, and using a partial budget model, Alvåsen et al. presented an analysis on animal welfare and the economic aspects of using nurse sows for equalizing the number of piglets per nursing sow in Sweden. They found that the lactation period of sows in Sweden is longer than in other countries, which can negatively affect sow body condition, damage teats and result in shoulder ulcers. Under nursing management practices, the piglet mortality rate could be reduced and higher financial returns generated, but the separating and mixing of piglets is stressful for piglets.

Hagerman et al. employed and assessed three estimation techniques for determining the value of replacement beef cows under data availability constraint, namely: hedonic pricing, vector error correction modeling, and cost of production. After analyzing the performance of each of these livestock valuation techniques, the authors concluded that the selection of a valuation method might need to vary based on data availability and characteristics of the livestock being valued, in terms of quality and age.

## Economic and Policy Assessment of Contagious Transboundary Animal Diseases and Zoonoses

A study by Truong et al. focused on assessing the economic impact of FMD outbreaks in beef and dairy farms in Long An and Tay Ninh provinces in South Vietnam. The authors also evaluated the economic justification of a biannual vaccination strategy to prevent and eradicate FMD. Results showed that FMD vaccination had a better net present value in large dairy farms than in small ones and had a 20-times higher net present value in dairy farms than in beef farms. They concluded that a biannual vaccination strategy is economically justifiable in dairy farms, but there was uncertainty about its justification in beef farms. Featuring FMD as a serious threat for international trade, Feng et al. assessed sectoral-level impacts of control measures on FMD outbreaks in FMD-free countries using a partial equilibrium model of the agricultural sector known as FAPRI-UK. By combining epidemiologic and economic modeling frameworks, the authors simulated and assessed the consequence of two control strategies of “stamping out” and “vaccinate-to-die” on commodity market prices in the UK. Given the assumptions used, their analyses showed that the price and value of output impacts were lower under the “vaccinate-to-die” strategy compared to the “stamping out” strategy.

Focusing on another highly contagious disease control policy, Thuy Nguyen et al. conducted a stakeholder survey of live bird markets and assessed the impact of closure of these markets as a mitigation measure for HPAI in Viet Nam. Their analysis demonstrated that it is very likely that trading outside of formal markets will occur in the event of a temporary live animal market closure. Hence, the authors concluded that strict enforcement, engagement with stakeholders, and adequate communication are important prerequisites before market closure policy is introduced. By merging value chain analysis and participatory approaches to developing innovative tools for analyzing constraints to information flow, Antoine-Moussiaux et al. proposed a field-based perspective on value chain applications to HPAI prevention and control as an example of animal health systems within a One Health framework.

Munsick et al. assessed the costs and benefits of vaccinating individual sheep flocks against bluetongue virus using a stochastic simulation modeling approach with a representative rangeland sheep operation in the Big Horn Basin of the state of Wyoming in the United States. They compared the costs, benefit, and net impacts of both a killed virus vaccine and a modified-live virus vaccine under various outbreak scenarios. Results showed that a killed virus vaccine was not economically justifiable for producers in areas with a low probability of outbreaks. A modified-live vaccine has not been manufactured in Wyoming and needs rigorous authorization before legal use can start.

## Human Behavior in Relation to Animal Health and Market Analysis

Under our third category of papers, by using exploratory and then face-to-face interviews, Ciaravino et al. investigated the perceptions, opinions, attitudes, and beliefs of farmers and veterinarians influencing the effectiveness of the bovine tuberculosis (bTB) eradication programme in Spain. The authors demonstrated the value of qualitative research for assessing the effectiveness of health interventions that are influenced by social and behavioral factors rather than biological ones. Focusing on market behavior and drivers of live cattle prices in Central Cameroon, Motta et al. used a quantitative framework, namely a generalized additive mixed-effect model, to identify the factors contributing to cattle price. Their findings indicated that the age and sex of the cattle traded were important drivers of price along with local human and bovine population densities.

Overall, the collection of papers in this Research Topic provides a good read on a number of important aspects of socioeconomics of animal health and welfare. This reflects a fraction of the many valuable efforts at the global level that are devoted to improving our knowledge and filling existing gaps in the literature of the economics of animal health and welfare. The authors of this Editorial and the board of the ISESSAH have great hope that the research being conducted and presented in future Research Topics of ISESSAH as well as in other related initiatives will contribute to further closure of these knowledge gaps and support animal health policies.

## Author Contributions

BV drafted the editorial. BH had an advisory role and provided input at the designing stage of the Research Topic. JR, HH, and GG reviewed and revised the editorial and contributed to the reviewing and editing the papers published in the proceedings of the ISESSAH inaugural conference.

### Conflict of Interest

The authors declare that the research was conducted in the absence of any commercial or financial relationships that could be construed as a potential conflict of interest.
